# Neutralizing Efficacy of Encapsulin Nanoparticles against SARS-CoV2 Variants of Concern

**DOI:** 10.3390/v15020346

**Published:** 2023-01-25

**Authors:** Sara Khaleeq, Nayanika Sengupta, Sahil Kumar, Unnatiben Rajeshbhai Patel, Raju S. Rajmani, Poorvi Reddy, Suman Pandey, Randhir Singh, Somnath Dutta, Rajesh P. Ringe, Raghavan Varadarajan

**Affiliations:** 1 Molecular Biophysics Unit (MBU), Indian Institute of Science, Bengaluru 560012, India; 2 Virology Unit, Institute of Microbial Technology, Council of Scientific and Industrial Research (CSIR), Chandigarh 160036, India; 3 Mynvax Private Limited, 3rd Floor, Brigade MLR Centre, No. 50, Vani Vilas Road, Basavanagudi, Bengaluru 560004, India

**Keywords:** SpyTag, SpyCatcher, SWE adjuvant, Omicron, encapsulin, thermotolerant, negative stain, BA.4/5

## Abstract

Rapid emergence of the SARS-CoV-2 variants has dampened the protective efficacy of existing authorized vaccines. Nanoparticle platforms offer a means to improve vaccine immunogenicity by presenting multiple copies of desired antigens in a repetitive manner which closely mimics natural infection. We have applied nanoparticle display combined with the SpyTag–SpyCatcher system to design encapsulin–mRBD, a nanoparticle vaccine displaying 180 copies of the monomeric SARS-CoV-2 spike receptor-binding domain (RBD). Here we show that encapsulin–mRBD is strongly antigenic and thermotolerant for long durations. After two immunizations, squalene-in-water emulsion (SWE)-adjuvanted encapsulin–mRBD in mice induces potent and comparable neutralizing antibody titers of 10^5^ against wild-type (B.1), alpha, beta, and delta variants of concern. Sera also neutralizes the recent Omicron with appreciable neutralization titers, and significant neutralization is observed even after a single immunization.

## 1. Introduction

Coronavirus disease 2019 (COVID-19) is an ongoing pandemic that has caused more than 500 million infections and roughly 6 million deaths worldwide [1]. Active efforts have thus gone into vaccine development to curtail viral transmission and alleviate disease severity [2,3,4,5,6,7,8,9]. SARS-CoV-2 spike glycoprotein is the principal antigen present in vaccine formulations, due to its critical role in viral entry through the host angiotensin-converting enzyme 2 (ACE-2) receptor attachment and the subsequent fusion of viral and host cell membranes [10,11,12]. Consequently, new variants such as alpha (B.1.1.7), beta (B.1.351), delta (B.1.617.2), and Omicron (B.1.1.529) and its subvariants BA.2 and BA.4/5 have emerged by accumulating mutations in the spike, particularly in the receptor-binding domain (RBD), altering viral antigenicity and pathogenicity [13,14,15]. The emergence of these variants has severely affected the efficacy of existing vaccines, especially against Omicron [16,17,18]. Furthermore, challenges associated with cold-chain storage and the distribution of existing vaccines have been a barrier to vaccination in low-resource countries. Additionally, most existing vaccines necessitate multiple vaccinations to induce adequate levels of neutralizing antibodies. All these factors pose a hurdle in minimizing infections and associated viral evolution of SARS-CoV-2.

Advances in nanotechnology have enabled the development of improved vaccine candidates, capable of inducing potent antibody responses after a single immunization [19]. In this regard, self-assembling proteins are being increasingly explored as vaccine delivery modalities due to their ability to form multivalent interactions with host B-cells [20,21]. Ferritin and ferritin-like nanoparticles such as E2P, encapsulin, and two-component I53 nanoparticles have been widely utilized in developing vaccines against infectious diseases such as influenza and HIV [22,23,24,25]. We have previously displayed influenza hemagglutinin derived stem antigens on diverse self-assembling proteins such as 12 mer-MsDps2, 24-mer ferritin, and 180-mer encapsulin. Our nanoparticle antigens induced complete protection against homologous and heterologous challenge in mice [26]. Several SARS-CoV-2 nanoparticle vaccines have also been described recently, which display either a full-length spike or spike receptor-binding domain (RBD) [27,28,29]. In addition, many groups have shown the versatility of self-assembling nanocarriers in displaying multiple antigens simultaneously. Mosaic SARS-CoV-2 nanoparticles developed by Cohen et al. presenting RBDs from human and animal coronaviruses elicited cross-reactive immune response in mice [30]. Ferritin-based mosaic nanoparticles have also been developed for influenza, displaying hemagglutinins from two strains of the group 1 influenza virus which elicited neutralizing antibodies against a range of subtypes [31].

“Plug and display” strategies have also been explored in conjugation with self-assembling proteins, with SpyTag–SpyCatcher technology being especially useful [32]. SpyTag is a 13-residue peptide which forms a spontaneous isopeptide bond with its 115 amino acid long partner protein, SpyCatcher, resulting in stable covalently linked complexes [33]. We used the SpyTag–SpyCatcher conjugation system for facile conjugation of a monomeric receptor-binding domain derivative on self-assembling proteins available to us. We have previously reported the design of a ferritin-like nanoparticle, MsDPS2, displaying twelve copies of a receptor binding domain derivative (mRBD) through SpyTag–SpyCatcher chemistry [34]. This construct showed high immunogenicity but elicited subpar levels of neutralizing antibodies in mice immunization studies.

In the present study, we describe an improved nanoparticle-based SARS-CoV-2 vaccine immunogen using encapsulin—a self-assembling protein from *Myxcoccus xanthus* (PDB ID: 4PT2), as a scaffold for multivalent display of the monomeric receptor-binding domain derivative (mRBD). Our immunogen displayed 180 copies of the monomeric mRBD, which bound conformation-specific antibodies with high affinity. The particle was stable upon long term storage and, in mice, induced high titers of neutralizing antibodies even after a single immunization. Neutralizing antibody titers were enhanced by 42-fold after a single boost and neutralized alpha, beta, and delta variants of concern (VOCs) with IC_50_s similar to the wild type. Similar to other SARS-CoV-2 vaccines, there was a significant drop in neutralization titers against Omicron. However, the residual titers were well above the background.

## 2. Materials and Methods

### 2.1. Encapsulin–SpyTag and mRBD–SpyCatcher Construct Design

Encapsulin–SpyTag was designed by genetically fusing the 13 amino acid residue SpyTag (PDB: 4MLI) of the CnaB2 domain of *Streptococcus pyogenes* protein FbaB (accession number: Q8G9G1) to the C terminus of a single subunit of encapsulin (residues 1-287) (source: *Myxococcus xanthus*) (accession number: Q1D6H4) through a 21-residue linker (GSAGSAGSAGSAGASGSGESG). GS (Gly Ser) linkers were chosen due to their small size and ability to form hydrogen bonds with water, which provides flexibility as well as stability to the linkers [35]. The gene was in frame with a cleavable N-terminal 6x His tag for immobilized metal affinity chromatography (IMAC) Ni-NTA purification. This construct was cloned into the bacterial expression vector pET 28 a (+) under T7 promoter control. For mRBD-SpyCatcher construct design, SpyCatcher (residues 440–549) (PDB: 4MLI) of the aforementioned CnaB2 domain was genetically fused to the C terminus of previously described mRBD construct (residues 332–532) through an 8-residue linker (ASGSGGSGG). The construct was cloned into a mammalian expression vector pcDNA3.4 under the CMV promoter. To enable protein secretion, a tPA signal peptide was added at the N terminus of the gene. A cleavable 10× Histidine tag was fused at the C terminus through an HRV-3C precision protease cleavage site for Ni-NTA purification.

### 2.2. Protein Purification and Conjugation

mRBD–SpyCatcher was purified from transiently transfected Expi293F cells following the manufacturer’s guidelines (Gibco, Thermo Fisher, Waltham, MA, USA). Briefly, Expi293F cells were diluted to a density of 3 × 10^6^ cells/mL. For transfection, the desired plasmid was complexed with ExpiFectamine293 according to the manufacturer’s protocol and transiently transfected into Expi293F cells. Sixteen hours after transfection, Enhancer 1 and Enhancer 2 were added. The culture supernatant was collected five days post-transfection, and protein was purified through Ni-NTA affinity chromatography using Ni Sepharose 6 fast-flow resin (GE Healthcare, Chicago, IL, USA). The supernatant was diluted two-fold using 1× PBS (pH 7.4) and applied to a pre-equilibrated Ni-NTA column at 4 °C. Following a 10 CV wash with 1× PBS (pH 7.4) supplemented with 20 mM imidazole, the protein was eluted in a gradient of imidazole (100, 200, 300, 400, and 500 mM) in 1× PBS (pH 7.4). Pooled eluted fractions were dialyzed thrice against 1× PBS (pH 7.4) and concentrated up to 1 mg/mL.

Encapsulin–SpyTag was purified from *E. coli* Rosetta cells. Briefly, a primary culture was started with a single sequence confirmed colony and grown overnight at 37 °C under shaking conditions. This was followed by secondary inoculation of 1% of primary culture in 500 mL Terrific Broth media (Himedia Laboratories Pvt. Ltd., Mumbai, India), grown at 37 °C till an OD_600_ of 0.6–0.8 was reached. The culture was induced with 1 mM IPTG and grown for an additional 16–20 h at 16 °C under constant shaking. Cells were harvested and lysed by sonicating in ice in 1× PBS (pH 8) supplemented with 1 mM PMSF protease inhibitor. Cell supernatant was collected and applied to pre-equilibrated Ni-NTA resin (GE Healthcare) and eluted with a 100 mM to 500 mM imidazole gradient in 1× PBS (pH 7.4). Eluted fractions were pooled and concentrated up to 1 mg/mL.

Nanoparticle display was facilitated through conjugation and was optimized by varying incubation temperature and ratio of the two protein partners (Figure S3). Finally, the reaction was set up at 37 °C for 12 h. Encapsulin–SpyTag and mRBD–SpyCatcher were mixed in a 1:3 molar ratio in 1× PBS pH 7.4, whereas mRBD–SpyCatcher was kept in molar excess for efficient conjugation. The reaction mixture was monitored on 12% SDS PAGE for complex formation.

### 2.3. SDS PAGE, Size Exclusion Chromatography (SEC) and Size Exclusion Chromatography-Multiangle Light Scattering (SEC-MALS)

Protein purity and conjugation were assessed through SDS PAGE in reducing condition. Samples were prepared by boiling in Dithiothreitol containing SDS sample loading buffer.

SEC and SEC-MALS confirmed the oligomerization status and mRBD display on encapsulin nanoparticles. Purified, unconjugated, and conjugated proteins were eluted from an analytical Superose 6 Increase 10/300 column (GE Healthcare) in 1× PBS (pH 7.4) at a 0.5 mL/min flow rate on an Äkta pure chromatography system. The peak area under the curve was evaluated using the inbuilt peak integrate tool. For SEC-MALS, proteins were purified using SEC and applied to an in-line MALS (mini-DAWN TREOS, Wyatt Technology, Santa Barbara, CA, USA) with a refractive index monitor (WATERS) for molecular weight confirmation. As described previously, ASTRA 6.0 software (Wyatt Technology) was used to analyze the data [36].

### 2.4. Equilibrium Thermal Unfolding Using nanoDSF

Thermal melting studies of the conjugated protein were performed using nanoDSF (Prometheus NT.48), as described [37], with 2–4 µM of the protein in 1× PBS (pH 7.4) at a temperature range of 20 °C to 95 °C in two independent measurements. 50% LED power was used.

### 2.5. SPR Binding Studies of Encapsulin-mRBD to ACE2-hFc and CR3022

The kinetic binding profile of Encapsulin-mRBD to ACE2-hFc and CR3022 was followed on a ProteOn XPR36 Protein Interaction Array V.3.1 (Bio-Rad, Hercules, CA, USA) at 20 °C as described previously [34]. Protein G in 10 mM sodium acetate buffer (pH 4.5) was immobilized in desired channels at a flow rate of 30 µL/min on an NHS-EDC (Sigma, St. Louis, MO, USA)-activated GLM chip until ~3500–4000 RUs were achieved. The coupling reaction was quenched using 1 M ethanolamine. Subsequently, ACE2-hFc and CR3022 ligands in 1× PBS (pH 7.4) were immobilized at a flow rate of 30 µL/min at 800 RUs. One channel acted as a reference. Encapsulin-mRBD analyte was flown over ligand-immobilized sensor channels in a concentration series in PBS (pH 7.4) with 0.05% P20 surfactant at a flow rate of 30 μL/min for 200 s for the association phase, and the subsequent dissociation phase was monitored for 600 s. An empty lane without ligand immobilization was utilized for measuring nonspecific binding. Following each kinetic assay, regeneration was carried out with 0.1 M Glycine-HCl (pH 2.7). The kinetic parameters were obtained by fitting the data to a simple 1:1 Langmuir interaction model using Proteon Manager.

### 2.6. SPR Binding of Encapsulin-mRBD to ACE2-hFc after Thermal Stress

Encapsulin-mRBD in 1× PBS (pH 7.4) was subjected to transient thermal stress by incubating at various temperatures (20 °C, 30 °C, 40 °C, 60 °C, and 80 °C) in a thermocycler (Eppendorf, Hamburg, Germany) for 60 min. Following that, the protein was allowed to cool to RT, and binding to ACE2-hFc was monitored through SPR as described in the previous section. For binding studies after prolonged thermal stress, Encapsulin-mRBD in 1× PBS (pH 7.4) was incubated at 4 °C and 37 °C for 4 weeks. Binding to ACE2-hFc and CR3022 was recorded after 2 weeks and 4 weeks, using SPR, as described in the previous section.

### 2.7. Negative Staining Transmission Electron Microscopy (TEM) Sample Preparation and Data Collection

The overall homogeneity and particle distribution of the freshly conjugated encapsulin–mRBD complex was visually inspected using negative staining transmission electron microscopy (NS-TEM). Carbon-coated 400 mesh Cu TEM grids were glow-discharged in a GloQube glow discharge apparatus for 30s followed by application of 3.5 μL of 1 mg/mL encapsulin-mRBD sample. The sample was incubated on the TEM grid for 1 min at room temperature. Subsequently, the excess sample was gently removed with Whatman filter paper. One percent of freshly prepared uranyl acetate solution was used for negative staining. The data were acquired in a 120 kV Talos L120C room temperature electron microscope equipped with a bottom-mounted Ceta camera (2Kx2K) at 73,000× magnification at a calibrated pixel size of 3.68 Å/pixel at specimen level.

### 2.8. Negative Staining TEM Data Processing

The raw micrographs were imported into EMAN2.2 for downstream processing. Nearly 2488 encapsulin-mRBD particles were picked using swarm mode, and the coordinates were extracted with e2boxer.py in EMAN2.2 software [38]. Multiple rounds of reference-free 2D classifications were performed to curate the particles using simple_prime2D of SIMPLE 2.0 software [39].

### 2.9. Mice Immunizations

Female BALB/c mice (6–8 weeks old, approximately 16–17 g, n = 5/group) were immunized intramuscularly with 20 µg of encapsulin-mRBD (20 µg/animal/dose in 50 µL of 1× PBS (pH 7.4), 1:1 v/v antigen: adjuvant ratio) adjuvanted with SWE (Sepivac SWE Batch No. 200915012131, Cat. No. 80748J, SEPPIC SA) on days 0 (prime), and 21 (boost). One mouse died during the pre-prime bleed. Sera were isolated from remaining mice (n = 4) from blood drawn on days prior to prime (day −1), post-prime (day 14), and post-boost (day 35) through retro-orbital puncture. Serum from prime immunization from one mouse was limited in volume. Hence, neutralization assay and ELISA to estimate scaffold titer after prime immunization were carried out with sera from three mice. This study was performed at Central Animal Facility, Indian Institute of Science. The Institutional Animal Ethics committee approved all animal studies (IAEC no. CAF/ETHICS/847/2021).

### 2.10. ELISA-Serum Antibody Endpoint Titers

Serum antibody-binding titers were measured as described previously [26]. Briefly, mRBD or spike-2P (4 µg/mL, in 1× PBS, 50 µL/well) was coated on 96-well plates which were incubated at 25 °C for 2 h. Four washes with 1× PBST (200 µL/well) were given, following which wells were blocked with blocking solution (100 µL, 3% skimmed milk in 1× PBST) for 1 h at 25 °C. Post blocking, fourfold serial dilutions of antisera starting at 1:100 dilution were added to each well (60 µL/well), and plates were incubated for 1 h at 25 °C. Wells were washed thrice with 1× PBST (200 μL of 1× PBST/well). Finally, ALP enzyme-conjugated rabbit anti-mouse IgG secondary antibody (Sigma-Aldrich) (diluted 1:5000 in blocking buffer) (50 µL/well) was added, and incubated for 1 h at 25 °C. The plate was developed with pNPP liquid substrate (50 µL/well) (pNPP, Sigma-Aldrich). The chromogenic signal was measured at 405 nm after 30 min of incubation at room temperature using an ELISA plate reader (Maxome Labsciences Cat # P3-5x10NO). The serum dilution with a signal observed twofold above the negative control (empty blocked wells) was considered the endpoint titer for ELISA.

### 2.11. Competition ELISA

Sera’s ability to compete with monoclonal antibodies was evaluated through ELISA, as previously described [40]. Briefly, mRBD (4 µg/mL, in 1× PBS (pH 7.4), 50 µL/well) was coated on 96-well plates followed by overnight incubation at 4 °C under constant shaking. Ovalbumin-coated wells (4 µg/mL in 1× PBS, 50 µL/well) were used as negative controls for mRBD immobilization. Wells were washed thrice with 1× PBST and blocked using blocking solution (100 µL of 3% skimmed milk in 1× PBST) for 45 min at 25 °C at 300 rpm. Two-fold serially diluted mice antisera starting at 1:80 dilution in 1× PBS (60 µL/well) were added to the desired wells and incubated at 25 °C for 1 h at 300 rpm. Only blocking solution was added to control wells for no sera. Three additional washes were given with 1× PBST (200 µL/well). Subsequently, ACE2-hFc, antibody S309, antibody COVA2-15, or antibody CR3022 were added (60 µL/well at 20 µg/mL) to their respective wells in an excess amount and plates were incubated at 25°C, 300 rpm for 1 h. Plates were washed thrice (200 µL of PBST/well) to remove excess unbound proteins. Finally, alkaline phosphatase conjugated goat anti-human IgG antibody (Sigma-Aldrich, St. Louis, MO, USA, Cat # AP112A; diluted 1:5000 in blocking buffer, 50 µL/well) was added, and plates were incubated at 25 °C, 300 rpm for 1 h. Following four washes with 1× PBST (200 µL/well), 50 µL/well of a pre-warmed alkaline phosphatase yellow (pNPP) liquid substrate (Sigma-Aldrich, Cat # P7998) was added to each well and the chromogenic signal measured at at 405 nm on an ELISA plate reader (Maxome Labsciences Cat # P3-5x10NO) after 30 min of incubation at 37 °C, 300 rpm, as described in previous section.

The percent competition was calculated using the following equation:

% Competition = [Absorbance_(control (no sera))_ − Absorbance_(serum dilution)_]/(Absorbance_(control (no sera))_) × 100 (1)

where absorbance control (no sera) is the absorbance measured at 405 nm of the competing protein (ACE2-hFc, CR3022, S309, or COVA2-15) binding to mRBD in the absence of sera, and absorbance serum dilution is the absorbance measured from wells where the serum dilution is incubated with mRBD in the presence of ACE2-hFc, CR3022, S309, or COVA2-15. Percent competition was calculated by fitting the serum dilution factor and % competition data points using a built-in three-parameter non-linear least-square fit curve from GraphPad Prism 8.4.2. IC_50_ competition titer was defined as the sera titer at which 50% competition was observed in the fitted curve.

### 2.12. Pseudovirus Neutralization Assay

SARS-CoV-2 pseudovirus harboring Nanoluc luciferase gene was generated by transient transfection of pHIV-1 NL4.3Δenv-Luc and Spike-Δ19-D614G or Spike Δ19-D614G plasmids with VOC mutations plasmids in HEK293T cells using Profection mammalian transfection kit (Promega Inc., Madison, WI, USA) according to the manufacturer’s protocol. After 48 h, pseudoviruses were harvested from the culture supernatant (600× *g*, 10 min), filtered, and stored at −80 °C until further use. ACE2 and TMPRSS2 receptors expressing HEK293T cells were grown in 10% FBS (Fetal Bovine Serum) supplemented DMEM (Gibco) with penicillin-streptomycin (100 U/mL). Heat inactivated sera were serially diluted and mixed with pseudoviruses for 1 h at 37 °C. Finally, 1 × 10^4^ HEK293T-ACE2-TMPRSS2 cells were added and incubated for 48 h at 37 °C with 5% CO2. Post incubation, nano-Glo luciferase substrate (Promega Inc.) was added (50 µL) and 80 µL lysate was transferred to flat-bottom white plates for luminescence measurement using Cytation-5 multimode reader. Serum dilution at which 50% reduction in infection was observed was determined as the pseudovirus neutralization titer (ID_50_).

### 2.13. Statistical Analysis

Binding and neutralizing titers between groups were compared by two-tailed Mann–Whitney test in the GraphPad Prism software 9.0.0 (* indicates *p* < 0.05 and ns indicates not significant). VOCs pseudoviral neutralization titers for the same sera with different VOCs were compared using in-built non-parametric Kruskal–Wallis test with Dunn’s correction of GraphPad Prism software 9.0.0 (* indicates *p* < 0.05 and ns indicates not significant).

## 3. Results

### 3.1. Design and Characterization of Encapsulin–mRBD Nanoparticle Vaccine

**Figure 1:**
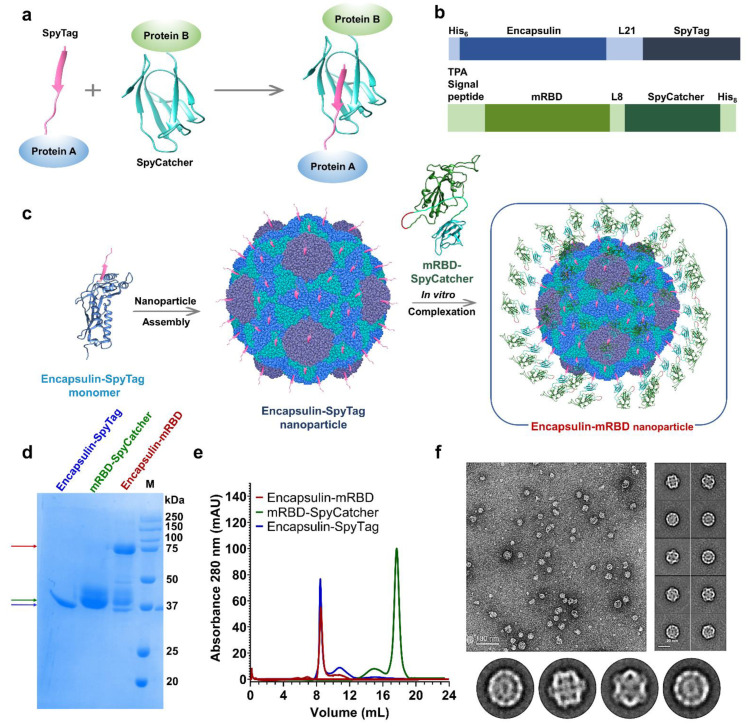
Design and biophysical characterization of Encapsulin-mRBD nanoparticles. (**a**), SpyTag–SpyCatcher chemistry. Shown here are two proteins, A and B, which are genetically linked to SpyTag and SpyCatcher, respectively. In vitro mixing of the two proteins results in spontaneous iso-peptide bond formation, leading to display of protein A on B. (**b**), Linear representation of the two conjugation partners, encapsulin–SpyTag and mRBD–SpyCatcher. Encapsulin (residues 1–287) (PDB id:4PT2) was genetically fused to a C terminal SpyTag through a 21-residue linker. A 6x-HisTag was attached at the N-terminus for Ni-NTA purification. mRBD (residues 332–532) was genetically linked to a C-terminal SpyCatcher via an 8-residue linker, as described previously [34]. An N-terminal TPA signal peptide and a C-terminal HisTag were attached for mammalian cell expression and Ni-NTA purification, respectively. (**c**), Schematic of SpyTag–SpyCatcher mediated conjugation of monomeric mRBD to Encapsulin nanoparticle. Genes in (**b**,**c**) are color coded for the ease of visualization as Encapsulin: blue, SpyTag: navy blue, monomeric mRBD: green, SpyCatcher: navy green. (**d**), Reducing SDS PAGE showing Encapsulin–mRBD conjugation. Lane 1—Unconjugated Encapsulin–SpyTag (monomeric MW: 37 kDa), Lane 2—Three-fold molar excess of mRBD–SpyCatcher (monomeric MW: 38 kDa), Lane 3—Conjugated Encapsulin–mRBD after overnight incubation at 37 °C (monomeric MW: 75 kDa) (Blue arrow denotes Encapsulin–SpyTag, green arrow denotes mRBD–SpyCatcher, red arrow denotes Encapsulin–mRBD). Lane 4—SDS PAGE protein ladder (M). (**e**), SEC profile of Encapsulin–mRBD (red), Encapsulin–SpyTag (blue) and mRBD–SpyCatcher (green). (**f**), Negative stain raw micrograph showing the distribution of Encapsulin–RBD oligomers (top left) (scale bar: 100 nm), and reference-free 2D class averages representing diverse orientations of the conjugated Encapsulin–mRBD complex (top right) (scale bar: 20 nm). Bottom panel shows enlarged views of representative negative staining TEM 2D class averages.

Encapsulins are iron-storing proteins present in bacteria capable of autonomous assembly into icosahedral complexes [41]. Each protomer of the Encapsulin can be fused to a desired antigen for surface presentation [42,43]. We have characterized Encapsulin for the display of Influenza hemagglutinin stem derived vaccine antigens, which provided complete protection against lethal challenge in mice [26]. In this case, the antigen of interest was a mammalian expressed, monomeric derivative of the SARS-CoV-2 spike receptor binding domain, mRBD (residues 332–532) [44]. We hypothesized that attachment of the mRBD to a large scaffold such as encapsulin may improve its immune detection and induce enhanced neutralizing antibody titers, compared to the monomer. The SpyTag–SpyCatcher system (ST-SC) was used for attachment of the mRBD to encapsulin (Figure 1a). This method offered two main advantages: spontaneous conjugation of the SpyTag–SpyCatcher upon mixing enabled facile display and avoided misfolding dependent aggregation that may arise from genetic fusion of large proteins. In addition, the ST-SC system also allows for creation of mosaic molecules which can display two or more antigens simultaneously and also for rapid assessment of different antigens conjugated to the same scaffold without the necessity to make multiple genetic fusions. To this end, we created encapsulin-SpyTag nanoparticles by genetically fusing each monomer of the 180-mer encapsulin (residues 1–287) (PDB id: 4PT2) to a C-terminal, 13-residue SpyTag (residues 550–562 of FbaB) (PDB id: 4MLI). A 21-residue linker was chosen to connect the two proteins to avoid steric clashes between neighboring SpyTags on the encapsulin surface and ensure complete conjugation of each encapsulin-SpyTag protomer. For conjugation to the nanoparticle, SpyCatcher (residues 440–552 of FbaB) (PDB id: 4MLI) was attached at the C-terminus of the mRBD through an 8-residue linker. Both proteins contained cleavable His-tags for immobilized metal affinity chromatography (IMAC) purification using Ni-NTA resins (Figure 1b,c). Encapsulin-SpyTag nanoparticles were purified from *E. coli* with a yield of ~21 mg/L and analyzed by SDS-polyacrylamide gel electrophoresis (SDS-PAGE) and size-exclusion chromatography multiangle light scattering (SEC-MALS) confirming protein purity and oligomerization (Figure S1a,c). mRBD–SpyCatcher was transiently expressed and purified from Expi293F suspension cells with a yield of ~80 mg/L and protein purity, and homogeneity was confirmed through SDS-PAGE and SEC-MALS (Figure S1b,d). A three-molar excess mRBD–SpyCatcher was added to the multimeric Encasulin–SpyTag for in vitro co-assembly, resulting in homotypic Encapsulin–mRBD nanoparticles that displayed 180 copies of the monomeric mRBD on their surface. Reducing SDS PAGE and size exclusion chromatography (SEC) of Encapsulin–mRBD revealed approximately 90 per cent conjugation and monodispersed particles (Figure 1d,e). The molecular mass was estimated from SEC-MALS to be 1.38 ± 0.009 × 10^7^ Da, confirming the formation of a 180-mer complex (Figure S2). Negative stain electron microscopy images of encapsulin–mRBD showed formation of icosahedral particles in solution, however, mRBD-SpyCatcher density on the particle surface could not be captured due to its ~1000-fold smaller size, relative to the encapsulin–SpyTag nanoparticle and the 21-residue long flexible linker resulting in an averaging out of mRBD–SpyCatcher’s density during the 2-D classification (Figure 1f). Nonetheless, SDS PAGE and SEC-MALS observations confirmed Encapsulin-mRBD conjugation and nanoparticle assembly.

### 3.2. Antigenicity and Thermal Stability of Encapsulin-mRBD Nanoparticle Vaccine

**Figure 2:**
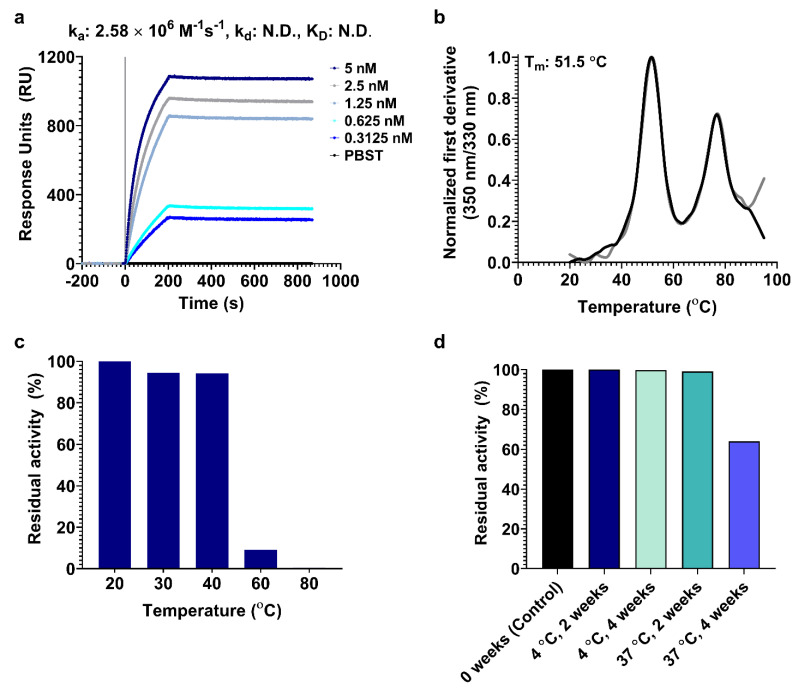
Antigenic characterization of Encapsulin-mRBD nanoparticles. (**a**), SPR-binding kinetics of encapsulin–mRBD to human ACE-2 receptor Fc region (ACE2-hFc). Five concentrations of two-fold serially diluted encapsulin–mRBD nanoparticles in 1× PBS (pH 7.4) were flowed at 30 μL/min. Ligands were immobilized in five different channels of the chip. A blank channel without any immobilization acted as reference, shown here as PBST (black). Encapsulin–mRBD concentration used: 5 (dark blue), 2.5 (grey), 1.25 (indigo), 0.625 (cyan), and 0.3125 nM (light blue). PBS (black). The kinetic parameters were obtained by fitting the data to a simple 1:1 Langmuir interaction model using Proteon Manager. (**b**), Thermal melt profile of conjugated encapsulin–mRBD nanoparticles in 1× PBS (pH 7.4), using nanoDSF. The experiment was carried out in duplicate, and both the traces are shown. (**c**), Thermal tolerance assay. Encapsulin–mRBD nanoparticle binding to ACE2-hFc was monitored through SPR after transient incubation at different temperatures (20, 30, 40, 60, and 80 °C) for 60 min in 1× PBS (pH 7.4) and subsequent cooling to room temperature. (**d**), Long term stability of encapsulin–mRBD nanoparticles, in 1× PBS (pH 7.4), was monitored by binding to ACE2-hFc using SPR after storage at 4 °C and 37 °C for 4 weeks.

To verify the antigenicity of Encapsulin–mRBD nanoparticles, we examined their reactivity with human ACE2-hFc fusion protein and a cross-neutralizing antibody, CR3022 [45], which targets a cryptic epitope on the outer side of the RBD through surface plasmon resonance (SPR) [45]. Encapsulin–mRBD bound both tested ligands with high association constant (k_a_) (ACE2-hFc: 2.58 × 10^6^ M^−1^s^−1^, CR3022: 5.44 × 10^6^ M^−1^s^−1^) and negligible dissociation, demonstrating that Encapsulin–mRBD adopted the spike RBD conformation (Figure 2a and Figure S4). mRBD nanoparticles exhibited a higher affinity for both the ligands, in comparison to the previously designed monomeric mRBD (K_D (ACE2-hFc)_: 14.2 nM and K_D (CR3022)_: 16 nM) showing that nanoparticle-based display of antigens facilitates multivalent and tighter binding to their cognate partners, compared to monomeric antigens [44].

We also evaluated the stability and cold-chain susceptibility of Encapsulin–mRBD particles. Encapsulin–mRBD (T_m_: 51.5 °C) exhibited comparable thermal stability to the monomeric mRBD (Tm: 50.3 °C) (Figure 2b) [44]. To evaluate the thermal tolerance of Encapsulin–mRBD, we analyzed its binding to Ace2-hFc after incubating in a range of temperatures for 60 min. Encapsulin-mRBD nanoparticles remained functional up to 40 °C in solution (Figure 2c). We next examined the longitudinal stability of Encapsulin-mRBD particles in solution by incubating them at 4 °C and 37 °C for four weeks and analyzing their binding to Ace2-hFc at different time points. Encapsulin—mRBD binding to Ace2-hFC was unaffected after storage at 4 °C for four weeks. At 37 °C, binding to Ace2-hFC was reduced by ~1 and ~36 per cent after two and four weeks of incubation, respectively (Figure 2d and Figure S5). Encapsulin–mRBD nanoparticles also retained the CR3022 epitopes upon storage under similar conditions (Figure S5). These results indicate stability of Encapsulin–mRBD nanoparticles in solution, which is a desirable feature for vaccine storage and last-mile distribution.

### 3.3. Immunogenicity of Encapsulin-mRBD Nanoparticle Vaccine in Mice

**Figure 3:**
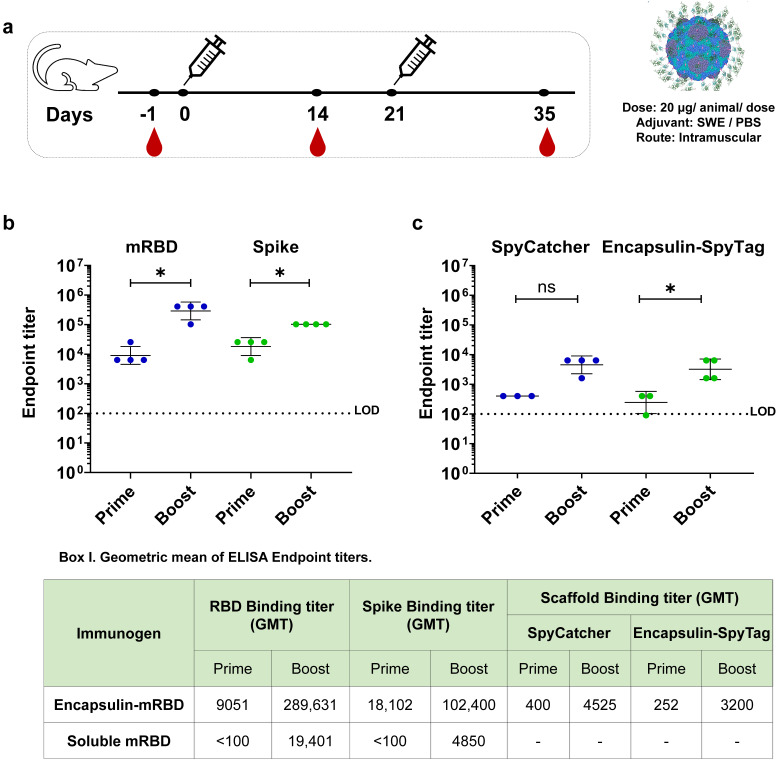
Immunogenicity of encapsulinm–RBD nanoparticles in mice. (**a**), Immunization scheme. (**b**), ELISA endpoint titers of encapsulin–mRBD nanoparticle elicited antisera against mRBD (blue) and spike protein (green) two weeks after prime and boost immunizations. (**c**), ELISA endpoint titers in the sera elicited by encapsulin–mRBD against the scaffold proteins–SpyCatcher and encapsulin–SpyTag measured two weeks after prime and boost immunizations. Binding antibody titers between groups were compared using two-tailed Mann–Whitney tests (* indicates *p* < 0.05 and ns indicates not significant). LOD is limit of detection. Geometric mean titers (GMT) of serum antibodies binding each protein two weeks after each immunization are indicated in Box I.

To characterize the immunogenicity of the mRBD–nanoparticles, BALB/c mice were immunized with either squalene-in-water emulsion (SWE) adjuvanted encapsulin–mRBD particles or PBS (control) at days 0 (prime) and 21 (boost) (Figure 3a). SWE is identical to Addavax and the MF59 oil in water emulsion adjuvant [46]. We have previously observed that both Addavax and SWE show similar immunogenicity in mice [34]. Humoral antibody response was evaluated for the sera collected from immunized mice two weeks after each immunization by enzyme linked immunosorbent assay (ELISA) against mRBD or spike. As expected, sera from mice immunized with PBS did not elicit quantifiable titers of antibodies after the first and second immunizations. Encapsulin–mRBD nanoparticles elicited high levels of mRBD and spike-binding antibodies after the first immunization. These titers were substantially higher relative to previous immunizations with the monomeric mRBD [40], consistent with improved humoral immune response against nanoparticle-based immunogens relative to their soluble counterparts [40]_._ RBD and spike-binding titers were significantly increased by ~32-fold (*p*-value: 0.02) and ~5.7-fold (*p*-value: 0.02), respectively, after the second immunization (Figure 3b). To address concerns of undesirable antibody titers against the scaffold, we evaluated antibody response against SpyCatcher and encapsulin–SpyTag after each immunization. Both SpyCatcher and Encapsulin–SpyTag specific antibody titers were significantly lower at all time points compared to the RBD (*p*-value: 0.02) (Figure 3c). Thus, multimerization of the mRBD strongly augmented its immunogenicity even after a single immunization, which enhanced manifold after a single boost. Together with low scaffold titers, these results highlight the display efficiency and robustness of the encapsulin–mRBD nanoparticle vaccine.

**Figure 4:**
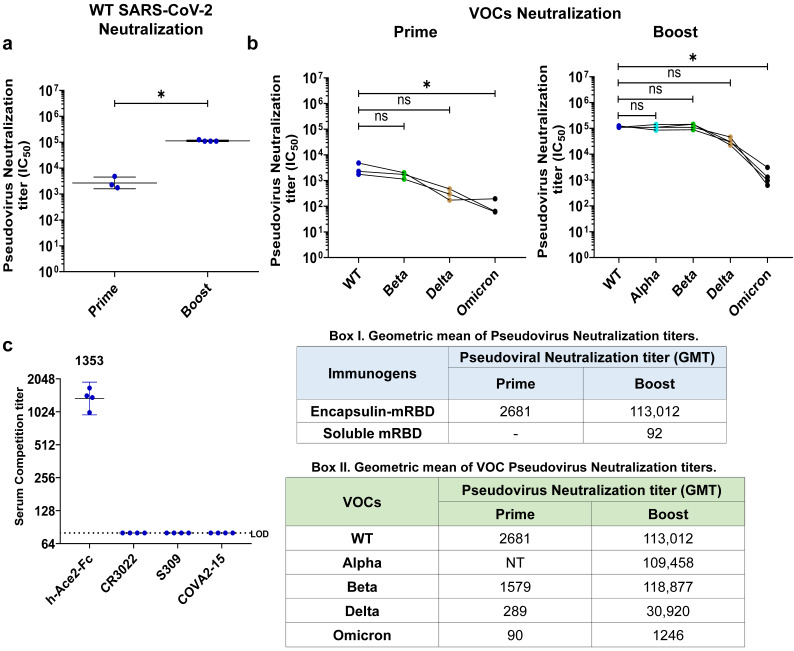
Neutralizing antibody response induced by encapsulin–mRBD nanoparticles in mice. (**a**), Pseudoviral neutralization titer (IC_50_) in sera elicited by encapsulin–mRBD nanoparticles measured two weeks after prime and boost immunizations. Neutralizing antibody titers between groups were compared using two-tailed Mann–Whitney tests (* indicates *p* < 0.05 and ns indicates not significant). (**b**), Pseudovirus neutralization titers elicited by encapsulin–mRBD nanoparticles against variants of concern (VOCs) evaluated two weeks after prime and boost immunizations. Statistical significance of differences between pseudovirus neutralization titers for different VOCs were analyzed through Kruskal–Wallis test using Dunn’s correction (* indicates *p* < 0.05 and ns indicates not significant). IC_50_ is defined as the sera dilution at which 50% neutralizing activity was observed. Geometric means of pseudovirus neutralization titers measured after prime and boost immunizations are indicated in Box I. Geometric mean of VOC pseudovirus neutralization titers measured after prime and boost immunizations are indicated in box II. NT indicates not tested. (**c**), ELISA titers (ID_50_) of serum antibodies competing with mAbs—ACE2-hFc, CR3022, S309 and COVA2-15 for binding to overlapping epitopes on the vaccine antigen, mRBD. ID_50_ is defined as the sera dilution at which 50% competition was observed. LOD is limit of detection.

We next examined the neutralizing activity of encapsulin–mRBD nanoparticles immunized sera using a panel of SARS-CoV-2 spike pseudotyped viruses. After the prime immunization, encapsulin–mRBD induced appreciable titers of neutralizing antibodies that neutralized the wild-type (WT) and beta variant of concern (VOC) to similar extents (*p*-value: > 0.99) and the Delta VOC with slightly lower neutralization titers, although, the decrease was not significant (*p*-value: 0.12). Sera also showed neutralization of the divergent Omicron BA.1, although with significantly reduced titers compared to WT (*p*-value: 0.01). Following the boost, neutralizing antibody titers increased significantly (*p*-value: 0.02). The reciprocal serum half-maximal inhibitory concentrations (IC_50_) for WT, beta, and delta were enhanced ~42, ~75, and ~107-fold, respectively. Post-boost sera neutralized the alpha and beta VOCs with IC_50_ comparable to the WT and the delta variant with ~3.65-fold lower IC_50_ (*p*-value: 0.23). Omicron neutralizing activity, although lower compared to WT, was also substantially boosted compared to prime (Figure 4a,b). When compared to previous immunizations with the monomeric RBD [40], encapsulin–mRBD nanoparticles induced neutralizing responses of increased breadth and magnitude.

To map the spike epitopes targeted by the sera, we performed competition ELISA with a panel of monoclonal antibodies—Ace2-hFc, COVA2-15 (Class 2), S309 (Class 3), and CR3022 (Class 4) for binding to RBD. COVA2-15 is a class 2 neutralizing antibody which targets an epitope partially overlapping with the Ace-2 binding region [47]. S309 and CR3022 are class 3 and 4 antibodies, respectively, with epitopes distal from the receptor binding motif and show broad Sarbecovirus neutralizing ability [45,48]. In addition, S309 and CR3022 are known to show cross neutralization of various SARS-CoV-2 variants, including Omicron [49,50,51]. Sera competition with any of these mAbs gives a measure of the breadth of epitopes targeted by the antibodies generated, and thus their cross-neutralizing ability. Although encapsulin–mRBD-induced sera neutralized a panel of VOCs including Omicron with appreciable titers, competition was only observed with Ace2-hFc. These results indicate that encapsulin–mRBD elicited sera mediated neutralization by blocking Ace-2 binding (Figure 4c). Overall, these findings indicate that nanoparticle display of the mRBD resulted in an efficacious vaccine design which elicits high neutralizing antibody titers against circulating VOCs, including Omicron.

## 4. Discussion

Since its outbreak in late 2019, SARS-CoV-2 spread globally, infecting millions of people, and was soon declared a pandemic. Global regulatory bodies approved 21 vaccines for emergency use, 6 of which were authorized by World Health Organization (WHO) including BNT162b2 (Pfizer), mRNA-12773 (Moderna), AZD1222 (Astra Zeneca), Ad26.COV2.S (Janssen), and NVX-COV2373 (Novavax) [52], and which showed remarkable efficacy against ancestral SARS-CoV-2. However, with the advancement of the pandemic, new variants with reduced sensitivity to these vaccines have emerged, raising serious concerns over the efficacy of existing vaccines against circulating and emerging variants. Recent studies have shown reduced neutralizing activity in the vaccinated or convalescent sera against VOCs, especially beta and Omicron [53,54,55,56,57]. Increasing cases of vaccine breakthrough infections by delta and Omicron variants have also been reported evidencing the inability of current vaccines in mounting sufficient protective responses against these variants [58,59,60]. Although clinical studies are evaluating the efficacy of booster doses in preventing infection [61,62,63], administration of multiple doses is cumbersome and costly. Therefore, alternative vaccine strategies such as antigen optimization and multimerization are being explored.

Several studies have shown the potential of nanoparticle-based vaccines in eliciting robust titers of long-lasting protective antibody response. A SARS-CoV-2 ferritin nanoparticle vaccine displaying an engineered spike derivative elicited humoral and T cell response in rhesus macaques and induced neutralization of SARS-CoV-2 variants as well as SARS-CoV-1 [64]_._ In another study, mice immunized with 0.5 or 0.1 μg of SARS-CoV2-2 RBD displayed on SpyCatcher-mi3 nanoparticles induced high titers of neutralizing antibodies that were superior to convalescent sera [65], demonstrating the neutralizing potency of nanoparticle vaccines even at low doses. Mosaic nanoparticle vaccine candidates co-displaying mutant SARS-CoV-2 spikes have also been developed that induced neutralization of heterologous strains in mice and cynomolgus macaques [30,66]. However, many factors, such as nanoparticle-based antigen safety, longterm stability, and efficacy, need to be thoroughly investigated before advancement into clinical trials. In the present study, we evaluated the protective efficacy of 20 μg of adjuvanted encapsulin–mRBD nanoparticle vaccine against escape variants, in mice. We found that our nanoparticle vaccine elicited notably high titer of RBD specific antibodies after a single immunization which were enhanced ~32-fold after a single boost. The majority of these antibodies were Ace-2 blocking, and in pseudovirus neutralization assay, neutralized WT, beta, and delta VOCs to similar extents. After a single boost, pseudoviral neutralization titers elicited by encapsulin–mRBD nanoparticle vaccine increased to ~10^5^ and compared favorably to the reported post-boost pseudoviral neutralization titers induced by 20 μg of mRNA-1273 and 5 μg of BNT162b1 mRNA vaccines in mice [6,8,67,68]. While sera from vaccinees immunized with Pfizer and Moderna vaccines have reported a 10.3-fold and 12.4-fold drop in neutralizing activity against the beta variant, respectively, sera from encapsulin–mRBD nanoparticle-immunized mice neutralized the beta spike pseudotyped virus with ~1.05-fold higher titers compared to WT [54,69]. Although direct comparison is difficult due to differences is assay setup, time points, animal models, and vaccine formulations, in mice immunizations encapsulin–mRBD nanoparticles seemed to perform remarkably well in terms of VOC neutralization relative to contemporary vaccines.

When developing nanoparticle-based vaccines, scaffold-elicited titers are an important aspect to evaluate, however, they are often overlooked or not reported. In our case, we found that encapsulin–mRBD nanoparticles elicited very low levels of scaffold-directed antibodies and the ratio of antibodies in the sera targeting the antigen versus scaffold were 36 to 1 in the prime immunized sera and 91 to 1 in the boosted sera. These findings demonstrate the display efficiency and safety of the encapsulin–SpyTag platform developed by us for antigen presentation. The usage of SpyTag–SpyCatcher system for antigen conjugation to the nanoparticle allows for facile simultaneous display of multiple antigens, as has been previously explored in the case of I53 and mi3 nanoparticles for co-display of spike protein from SARS-CoV-2 variants or zoonotic coronaviruses [30,66]. However, for manufacturing, the necessity of a conjugation step and the presence of unligated material present potential complications. Hence, in the future, we will also compare the immunogenicity of mRBD genetically fused to encapsulin with the present Encapsulin–SpyTag–SpyCatcher–RBD nanoparticles.

The long-term stability of vaccines is pivotal for unaltered vaccine efficacy after storage and distribution. mRNA vaccines such as mRNA-1273 and BNT162b2 can be reportedly stored at 2–8 °C for up to 30 and 5 days, respectively, while protein subunit vaccine NVX-COV2373 can be stored at 2–8 °C for few weeks [35,67,68,69,70,71]. Extensive stability analysis revealed that Encapsulin–mRBD can be stored at 4 °C for four weeks and at 37 °C for up to two weeks without changes in antigenicity. In future, one could explore the use of a thermostable encapsulin scaffold to see if this results in improved thermal storage properties of the displayed fusion [70].

Although challenge studies were not undertaken in the present report, nanoparticle vaccines with pseudoviral neutralization titers of >10^2^ have shown protective responses in non-human primates with considerably reduced viral loads and lung pathology [71]. Our nanoparticle vaccine elicits ~100-fold higher titers of neutralizing antibodies, which are well above this threshold and thus are expected to show protection against SARS-CoV-2 challenge. Finally, we have shown the design of a robust platform for vaccine antigen display capable of eliciting superior neutralizing antibody titers compared to monomeric formulations, amenable to multiple antigen display and which elicits relatively low levels of undesirable scaffold directed titers, making it a promising candidate for future clinical development.

## Data Availability

The data presented in this study are contained within the article and Supplementary material.

## Supplementary Materials

The following supporting information can be downloaded at: https://www.mdpi.com/article/10.3390/v15020346/s1, Figure S1. Reducing SDS PAGE and SEC-MALS profile of purified protein before conjugation. Figure S2. SEC-MALS profile of Encapsulin-mRBD nanoparticles. Figure S3. Optimization of Encapsulin-mRBD conjugation through SpyTag–SpyCatcher chemistry. Figure S4. Antigenic characterization of Encapsulin-mRBD nanoparticles. Figure S5. Long term stability analysis of Encapsulin-mRBD nanoparticles.
