# Supplementary material for: Neutralizing Efficacy of Encapsulin Nanoparticles against SARS-CoV2 Variants of Concern
Source: Viruses. 2023 Jan 25;15(2):346. doi: 10.3390/v15020346 (PMC9961482; doi:10.3390/v15020346)
Supplement: Supplementary file 1 [file viruses-15-00346-s001.zip › viruses-2131548-supplementary/viruses-2131548-supplementary/Supplementary file_090223.pdf]

Supplementary Information

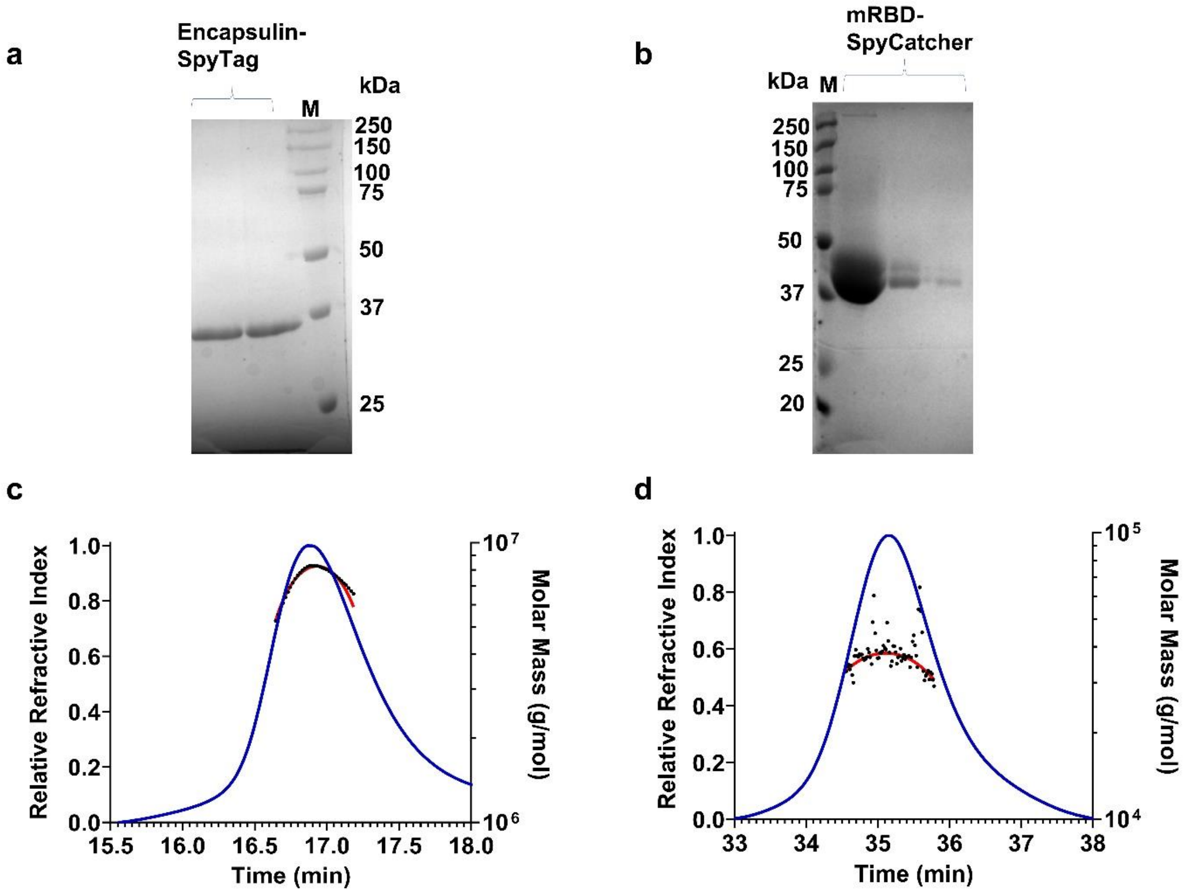

Figure S1. Reducing SDS PAGE and SEC-MALS profile of purified protein before conjugation. a-b, Reducing SDS PAGE image of purified **a**, Encapsulin-SpyTag (monomeric MW: 37 kDa) and **b**, mRBD-SpyCatcher (monomeric MW: 38 kDa). **c-d**, SEC-MALS profile of purified **c**, Encapsulin-SpyTag (oligomeric MW: 6.6 × 10^6 Da) and purified **d**, mRBD-SpyCatcher (monomeric MW: 38 × 10^3 Da). Proteins were separated on a Superose 6 Increase 10/300 column (GE Healthcare) in 1x PBS (pH 7.4) at a 0.5 mL/min flow rate on an Äkta pure chromatography system and analyzed through an in-line MALS (mini-DAWN TREOS, Wyatt Technology corp.) and Refractive Index detector (WATERS corp).

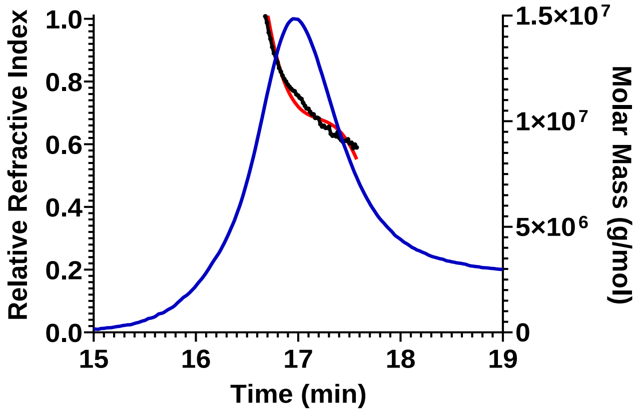

Figure S2. SEC-MALS profile of Encapsulin-mRBD nanoparticles. Protein was separated on a Superose 6 Increase 10/300 column (GE Healthcare) in 1x PBS (pH 7.4) at a 0.5 mL/min flow rate on an Äkta pure chromatography system and analyzed through an in-line MALS (mini-DAWN TREOS, Wyatt Technology corp.) and Refractive Index detector (WATERS corp). Molecular weight of Encapsulin-mRBD nanoparticles was estimated to be $1.38 \pm 0.009 \times 10^7$ Da.

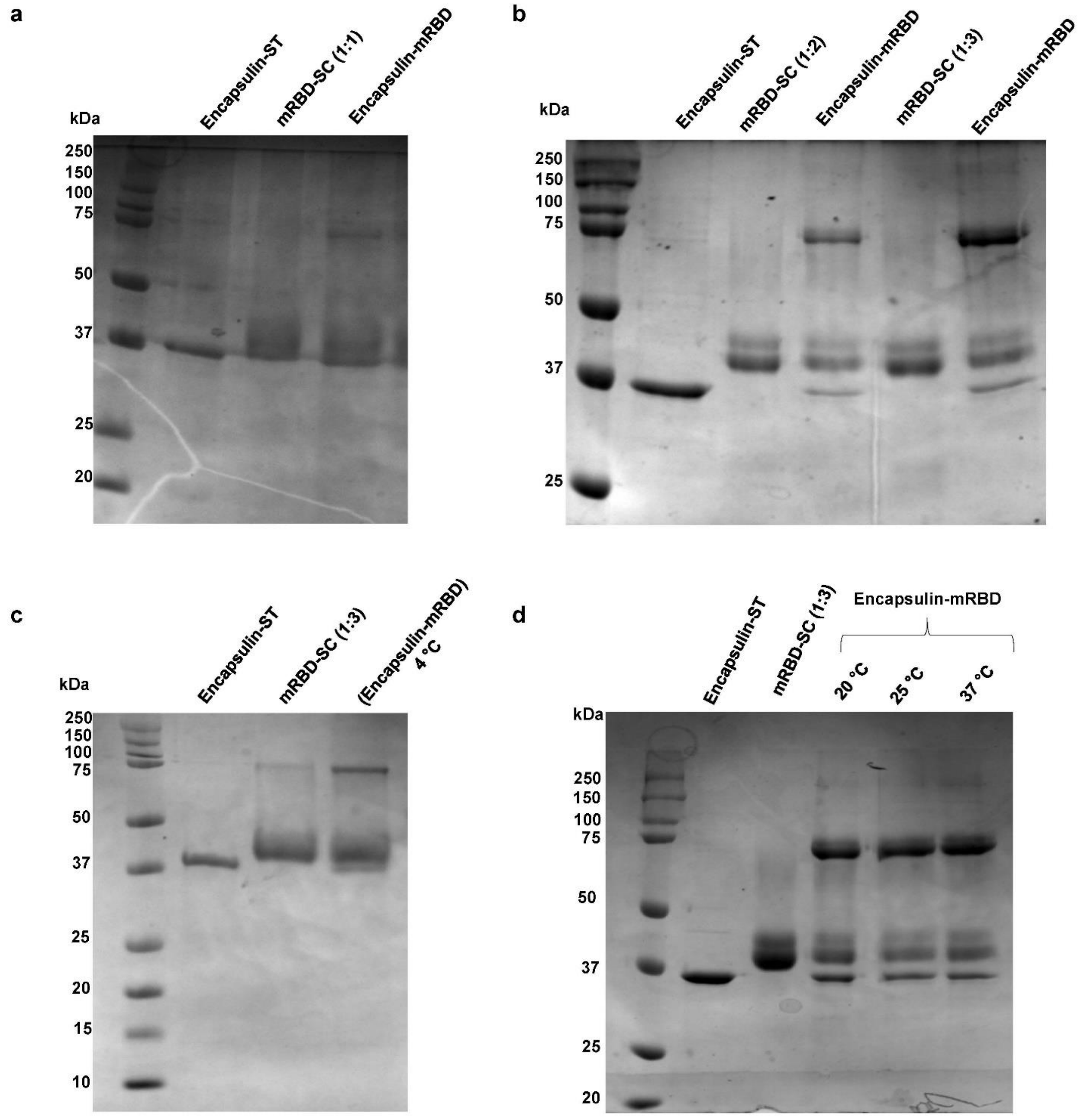

Figure S3. Optimization of Encapsulin-mRBD conjugation through SpyTag-SpyCatcher chemistry. a-b, Ratio optimization. Encapsulin-SpyTag was incubated with different molar ratios of mRBD-SpyCatcher. **a**, Encapsulin-SpyTag was incubated with mRBD-SpyCatcher in 1:1 molar ratio. **b**, Encapsulin-SpyTag was incubated with mRBD-SpyCatcher in 1:2 and 1:3 molar excess ratio. All reactions were carried out at 37 °C. **c-d**, Temperature optimization. Encapsulin-SpyTag was incubated with three molar excess mRBD-SpyCatcher at **c**, 4 °C, and **d**, 20, 25 and 37 °C.

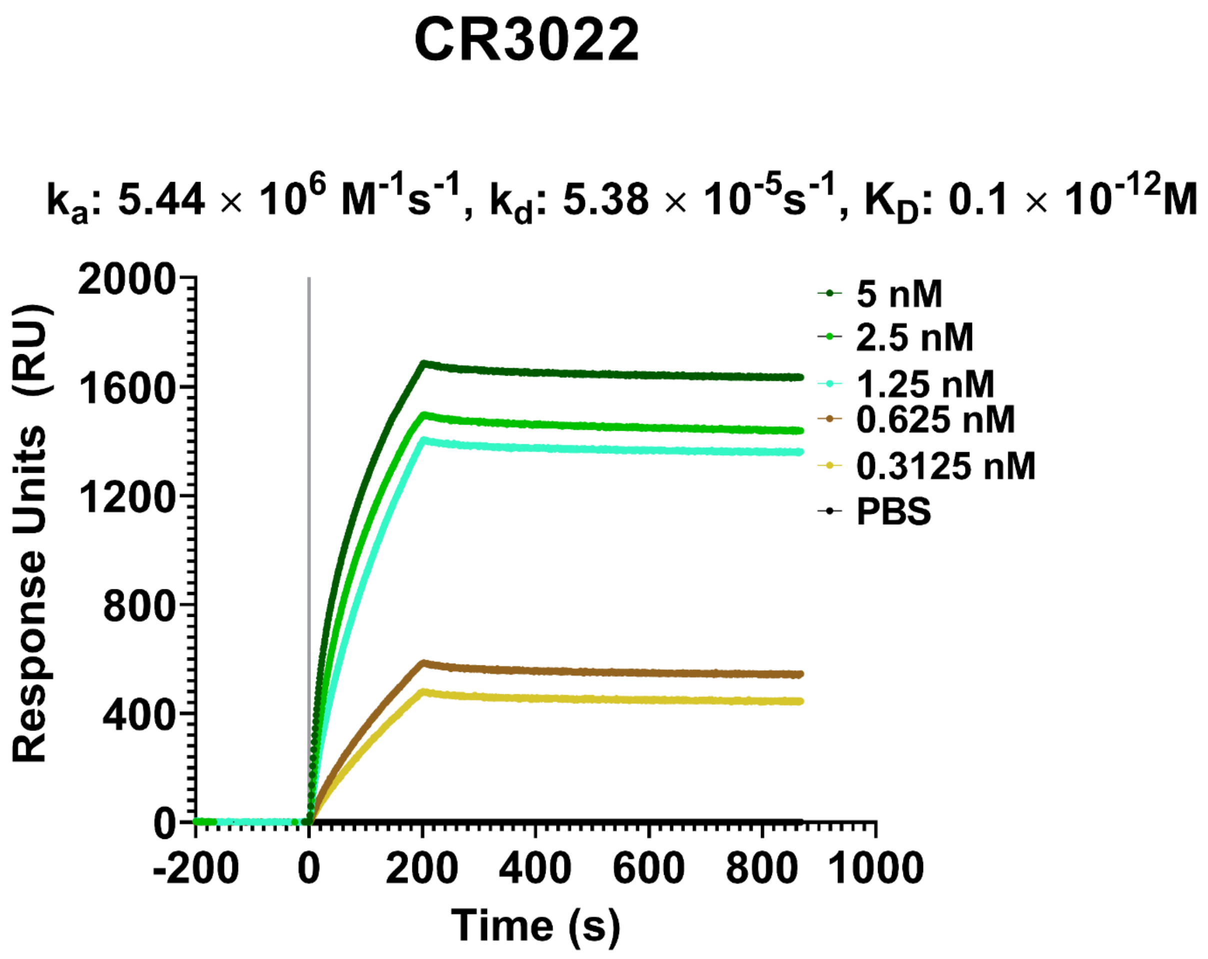

Figure S4. Antigenic characterization of Encapsulin-mRBD nanoparticles. Encapsulin-mRBD nanoparticle binding to RBD binding monoclonal antibody, CR3022, through SPR. Serial dilutions of Encapsulin-mRBD (5, 2.5, 1.25, 0.625, and 0.3125 nM) in 1x PBS (pH 7.4) were passed at a flow rate of 30 μL/min over a ligand immobilized CM5 chip. One channel was immobilized with CR3022, and one channel was left blank to act as reference (shown here as PBST). Binding traces were analysed by fitting into 1:1 Langmuir interaction model using Proteon Manager.

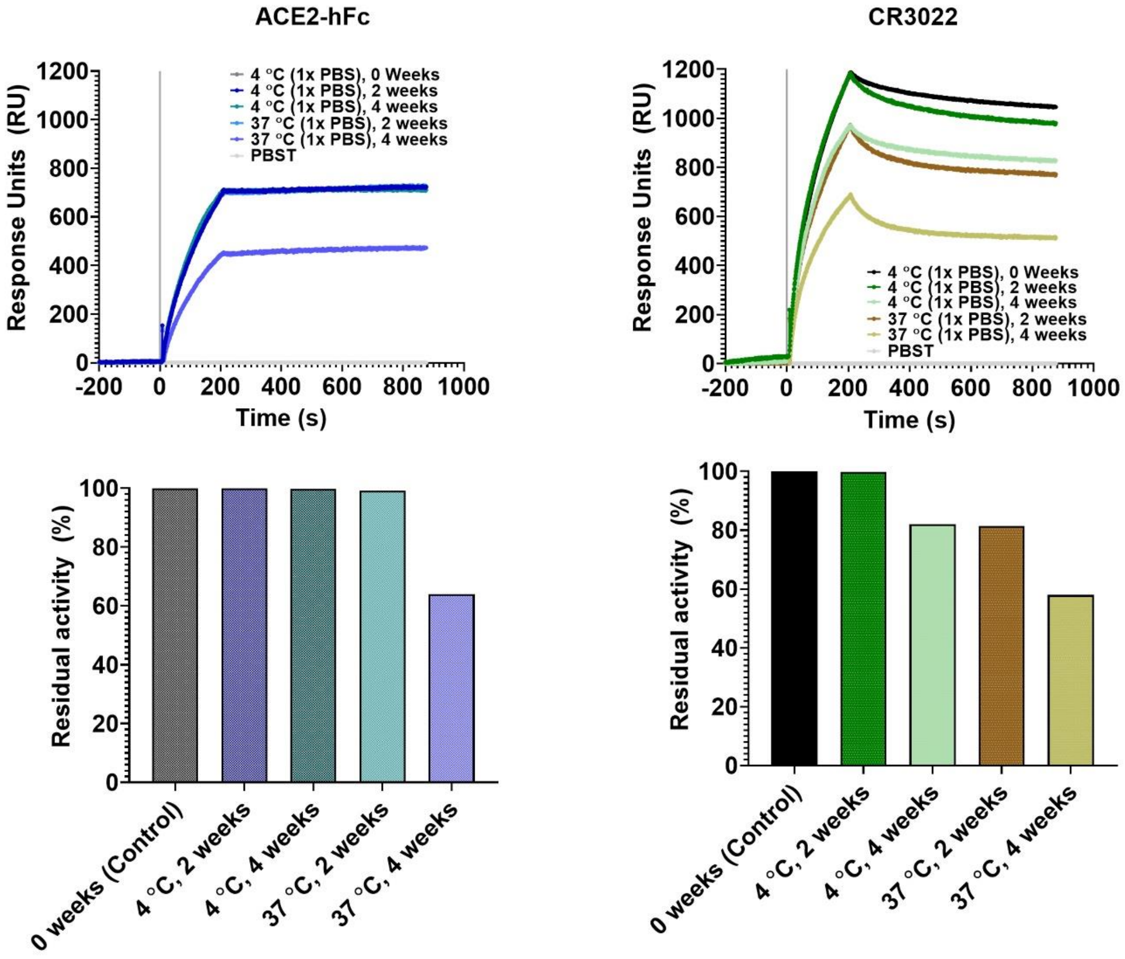

Figure S5. Long term stability analysis of Encapsulin-mRBD nanoparticles. Encapsulin-mRBD nanoparticles, in 1x PBS (pH 7.4), were incubated at 4 °C and 37 °C for four weeks. Binding to conformation specific ligands was examined after two and four weeks of storage through SPR. **a**, Binding of 5 nM of Encapsulin-mRBD nanoparticles to Ace2-hFC (top). Bar graph indicating residual binding activity (in percentage) to Ace2-hFc calculated taking 4 °C, 0 weeks as control for 100 per cent activity (bottom), as already shown in figure 2d. **b**, Binding of 5 nM Encapsulin-mRBD nanoparticles to monoclonal antibody, CR3022, after storage at indicated temperatures and timepoints (top). Bar graph indicating residual binding activity to CR3022, calculated as described above (bottom).
